# Effects of tolvaptan in patients with chronic kidney disease and chronic heart failure

**DOI:** 10.1007/s10157-016-1379-0

**Published:** 2017-02-11

**Authors:** Mari Katsumata, Nobuhito Hirawa, Koichiro Sumida, Minako Kagimoto, Yosuke Ehara, Yuki Okuyama, Megumi Fujita, Akira Fujiwara, Mayumi Kobayashi, Yusuke Kobayashi, Yuichiro Yamamoto, Sanae Saka, Keisuke Yatsu, Tetsuya Fujikawa, Yoshiyuki Toya, Gen Yasuda, Kouichi Tamura, Satoshi Umemura

**Affiliations:** 1 Department of Nephrology and Hypertension, Yokohama City University Medical Center, 45-7 Urafune-cho, Minami-ku, Yokohama, 232-0024 Japan; 2 Department of Medical Science and Cardiorenal Medicine, Yokohama City University School of Medicine, Yokohama, Japan; 3 Department of Nephrology, Yokosuka City Hospital, Yokosuka, Japan; 4 Center for Health Service Sciences, Yokohama National University, Yokohama, Japan; 5 Yokohama Rosai Hospital, Yokohama, Japan

**Keywords:** Chronic kidney disease, Tolvaptan, Urine osmolality, Hyponatremia

## Abstract

**Background:**

Tolvaptan, a vasopressin V_2_ receptor blocker, has a diuretic effect for patients with heart failure. However, there were a few data concerning the effects of tolvaptan in patients with chronic kidney disease (CKD).

**Methods:**

We retrospectively analyzed 21 patients with chronic heart failure and CKD. Tolvaptan was co-administered with other diuretics in-use, every day. We compared clinical parameters before and after the treatments with tolvaptan. Furthermore, we examined the correlations between baseline data and the change of body weight.

**Results:**

Tolvaptan decreased the body weight and increased the urine volume (*p* = 0.001). The urine osmolality significantly decreased throughout the study period. Urinary Na/Cr ratio and FENa changed significantly after 4 h, and more remarkable after 8 h (*p* = 0.003, both). Serum creatinine increased slightly after 1 week of treatment (*p* = 0.012). The alteration of body weight within the study period correlated negatively with the baseline urine osmolality (*r* = −0.479, *p* = 0.038), the baseline urine volume (*r* = −0.48, *p* = 0.028), and the baseline inferior vena cava diameter (IVCD) (*r* = −0.622, *p* = 0.017). Hyponatremia was improved to the normal value, and the augmentations of the sodium concentration were negatively associated with the basal sodium levels (*p* = 0.01, *r* = −0.546).

**Conclusions:**

Tolvaptan is effective in increasing diuresis and improved hyponatremia, even in patients with CKD. The baseline urine osmolality, urine volume, and IVCD may be useful predictors for diuretic effects of tolvaptan.

## Introduction

Tolvaptan is an oral diuretic with a new mechanism which selectivity binds to the V_2_ receptor as an antagonist. V_2_ receptors are located in the renal collecting duct, where arginine vasopressin (AVP) binding to the V_2_ receptor leads to a rise in intracellular cAMP. This promotes renal water reabsorption via translocation of intracellular vesicles containing the water channel aquaporin-2 into the apical plasma membrane and increased transcription of aquaporin-2 [1]. The compound’s aquaretic effects by single administration were confirmed by an increase in urine volume and a decrease in urine osmolality, followed by an increase in serum sodium concentration [2]. Tolvaptan has been demonstrated to ameliorate congestion in patients with heart failure [3, 4] and to correct hyponatremia [5, 6].

Patients with advanced CKD cannot produce enough urine and often fall into volume overload. Loop diuretics are conventionally used for these patients. However, there are several adverse effects with taking loop diuretics. One is electrolyte abnormalities, such as hypokalemia and hyponatremia [7]. On the other hand, tolvaptan has been reported to promote water excretion without changes in renal hemodynamics or sodium and potassium excretion [1].

However, the effects of tolvaptan on urine volume, and urinary and serum sodium excretions in advanced CKD patients were not yet clarified. Furthermore, the parameters which relate to body weight reduction were also not sufficiently evaluated. Thus, to clarify the effects of tolvaptan on body weight, and urinary and serum electrolytes, as well as to examine the predictors for efficacy, we retrospectively evaluated the medical records of CKD patients.

## Materials and methods

### Study subjects

This was a retrospective observational study. We included patients with CKD stage G3–5 and congestive heart failure, who were admitted to our hospital and received tolvaptan for excess body fluid, despite administration of the conventional diuretics between December 2010 and April 2015. The definition of CKD G3–5 was eGFR less than 60 ml/min/1.73 m^2^ over 3 months despite the underlying disease. We excluded the following: patients undergoing hemodialysis and peritoneal dialysis, those whose dose of other diuretics increased during the observation period (3 days before and a week after tolvaptan administration), and a case of acute kidney injury. The initial tolvaptan dose was 15 mg/day, except one case that started at 3.75 mg/day.

### Measurements

The main endpoints of this study were changes of body weight after 1 week administration of tolvaptan, and we compared average urine volume for 3 days before and for 7 days after treatment. In addition, we compared values of blood pressure, urine osmolality, urine gravity, urea sodium excretion calculated by spot urine (U-Na, mEq/gCr), fractional excretion of sodium (FENa), serum creatinine, estimated glomerular filtration rate (eGFR), serum sodium, B-type natriuretic peptide (BNP), human atrial natriuretic peptide (hANP), and antidiuretic hormone (ADH) before and after the administration of tolvaptan. We obtained data of urine osmolality, urine gravity, U-Na, and FENa after tolvaptan treatment at 4, 8, and 24 h. eGFR was calculated using the equation proposed by the Japanese Society of Nephrology as follows ; eGFR (ml/min/1.73 m^2^) = 194 × Cr^− 1.094^ × Age^−0.287^×0.739 (if female) [8]. We collected data of the ejection fraction and inferior vena cava diameter (IVCD) in the case that heart ultrasonography was performed before beginning tolvaptan. IVCD was visualized two-dimensionally and measured by M-mode echocardiography in expiration as described elsewhere [9]. In cases of discharge or drug interruption, the data at discontinuance were substituted.

### Statistical analysis

The value of each measurement was expressed as the median (interquartile range). Comparisons of parameters between before and after treatment were analyzed with the Wilcoxon signed-rank test. Factors correlating with changes of body weight and S-Na were analyzed with the Spearman rank correlation coefficient. We considered a *p* value <0.05 as significant. Data analysis was performed with SPSS 23 statistical software (SPSS Inc., Chicago, IL, USA).

## Results

Table 1 shows the clinical characteristics of the study subjects. The study consisted of 15 men and 6 women with a median age of 69 (interquartile range 60–76). Nineteen patients were in CKD stage 5; two patients were in stage 3a or 3b respectively. The median serum creatinine level just before administration of tolvaptan was 3.82 mg/dl (interquartile range 3.48–5.08). The average urine volume for 3 days before tolvaptan administration was distributed between 292 and 1900 ml/day, and the median of all cases was 975 ml/day. One patient was in NYHA IV and eleven patients were in NYHA III, and five were in NYHA II. Primary diseases included benign nephropathy (*n* = 10), diabetic nephropathy (*n* = 7), polycystic kidney disease (*n* = 1), microscopic polyangitis (*n* = 1), membranous nephropathy (*n* = 1), and lupus nephritis (*n* = 1). Patients were treated with the following diuretics: furosemide alone (median dose of 140 mg, interquartile range 80–200, *n* = 17), spironolactone 25 mg/day and trichlormethiazide 1 mg/day (*n* = 1); furosemide 40 or 320 mg/day and potassium canrenoate 400 mg/day (*n* = 2); or spironolactone alone (25 mg/day, *n* = 1). The renin–angiotensin–aldosterone system (RAAS) inhibitor was prescribed for 12 patients. Only 1 patient took digitalis.

**Table 1:** Clinical characteristics of patients

	Value
Age (median, IQR)	69 (60–76)
Sex (male) [*n* (%)]	15 (71.4)
Body weight (kg) (median, IQR)	62.6 (54.6–72.5)
Body mass index (kg/m^2^) (median, IQR)	24.8 (20.1–29.0)
Blood pressure (mmHg) (median, IQR)	
Systolic	136 (126–143)
Diastolic	74 (64–78)
NYHA [*n* (%)]	
I	4 (19.0)
II	5 (23.8)
III	11 (52.4)
IV	1 (4.8)
Primary disease [*n* (%)]	
Benign nephropathy	10 (47.6)
Diabetic nephropathy	7 (33.3)
Polycystic kidney disease	1 (4.8)
Microscopic polyangitis	1 (4.8)
Membranous nephropathy	1 (4.8)
Lupus nephritis	1 (4.8)
CKD stage [*n* (%)]	
G3a	1 (4.8)
G3b	1 (4.8)
G4	0 (0)
G5	19 (90.5)
Furosemide (mg/day) (median, IQR) (*n* = 19)	120 (60–200)
Use of ARB, ACEI [*n* (%)]	12 (57.1)
Ejection fraction (%) (median, IQR) (*n* = 15)	71.8 (59.6–76.9)
Inferior vena cava diameter (mm) (median, IQR) (*n* = 14)	15.7 (11.0–21.0)
Average urinary volume for 3 days before administration (ml/day) (median, IQR)	975 (783–1350)
Urine gravity (*n* = 19)	1.011 (1.008–1.013)
U-Na (mEq/gCr) (median, IQR) (*n* = 20)	99.2 (60.9–145)
FENa (%) (median, IQR) (*n* = 20)	3.13 (1.5–5.16)
Serum creatinine (mg/dl) (median, IQR)	3.82 (3.48–5.08)
eGFR (ml/min/1.73 m^2^) (median, IQR)	11.6 (9.2–13.9)
Serum sodium (mEq/l) (median, IQR)	139 (136–142)
Serum osmolality (mOsm/kg) (median, IQR) (*n* = 18)	299 (296–309)
Brain natriuretic peptide (pg/ml) (median, IQR)	488.9 (89.3–599.1)
Atrial natriuretic peptide (pg/ml) (median, IQR) (*n* = 13)	104.0 (50.8–163.0)
Antidiuretic hormone (pg/ml) (median, IQR) (*n* = 9)	2.9 (1.4–3.5)
Urine osmolality (mOsm/kg) (median, IQR) (*n* = 19)	313 (269–352)

*IQR* interquartile range, *n* number, *NYHA* New York association criteria, *CKD* chronic kidney disease, *ARB* angiotensin II receptor blocker, *ACEI* angiotensin converting enzyme inhibitor, *U-Na* urine sodium excretion, *FENa* fractional excretion of sodium, *eGFR* estimated glomerular filtration rate

**Fig. 1:**
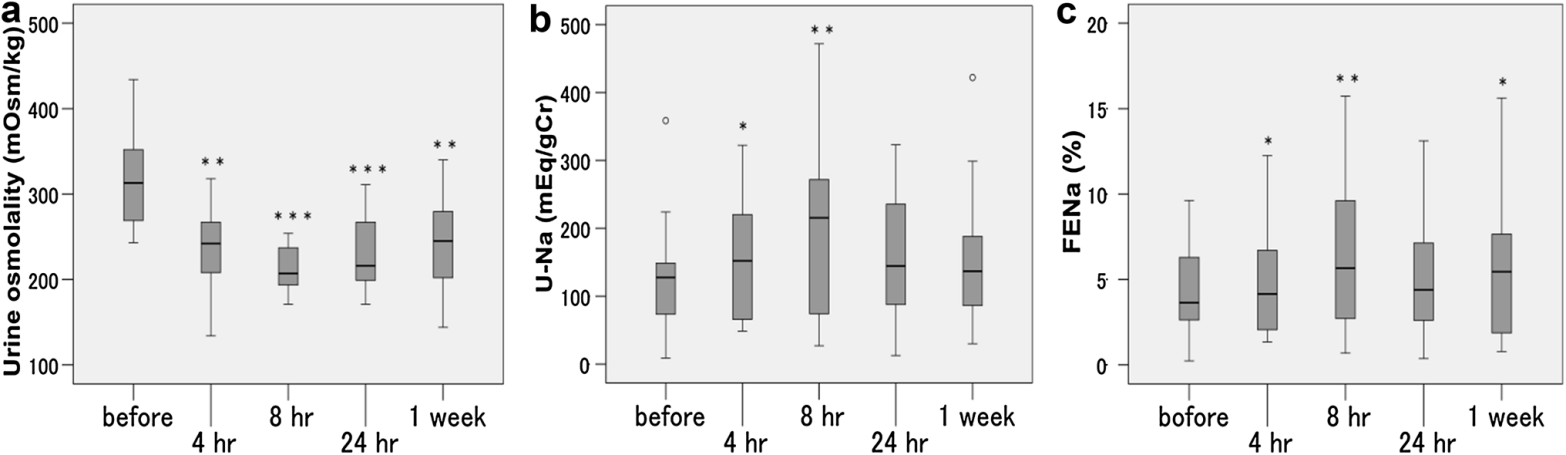
Alterations of urine parameters by tolvaptan treatment with CKD patients. **a** Urine osmolality decreases after 4 h (*n* = 17), and more remarkable at 8 h (*n* = 16). The effects on urine osmolality continued throughout the study periods. **b** Urinary sodium excretion (U-Na; mEq/gCr) increased significantly after 4 h (*n* = 17), and more remarkable after 8 h (*n* = 17). **c** FENa changed similar to U-Na, but also increased significantly 1 week later (*n* = 20). *p < 0.05, **p < 0.01, ***p < 0.001

Body weight decreased (*p* < 0.001), and urine volume increased with tolvaptan treatment (*p* < 0.001) (Table 2). Reflecting water diuresis, urine osmolality decreased significantly (Fig. 1a). The urine osmolality decreased after 4 h (*p* = 0.001), and markedly at 8 h (*p* < 0.001). The effects on urine osmolality continued throughout the study periods. The urine gravity significantly decreased 8 h (*p* = 0.043, *n* = 6) and 24 h (*p* = 0.002, *n* = 14), but not significant at 1 week (Table 2).

**Table 2:** Change of clinical parameters after administration of tolvaptan

Parameter	Baseline (median, IQR)	1 week (median, IQR)	*p* value
*Body weight (kg)*	**62.6 (54.6–72.5)**	**61.6 (47.7–70.9)**	**< 0.001**
*Average urinary volume (ml/day)*	**975 (783–1350)**	**1426 (1079–1557)**	**< 0.001**
Systric blood pressure (mmHg)	136 (126–143)	130 (119–147)	0.852
Diastolic blood pressure (mmHg)	74 (64–78)	68 (64–76)	0.794
Urine gravity (*n* = 19)	1.011 (1.008–1.013)	1.009 (1.008–1.012)	0.172
*Serum creatinine (mg/dl)*	**3.82 (3.48–5.08)**	**4.35 (3.74–5.45)**	**0.012**
*eGFR (ml/min/1.73 m* ^2^)	**11.6 (9.2–13.9)**	**9.8 (8.9–12.6)**	**0.008**
*Serum sodium (mEq/l)*	**139 (136–142)**	**141 (138–143)**	**0.002**
Brain natriuretic peptide (pg/ml)	488.9 (89.3–599.1)	337.0 (113.1–506.0)	0.117
Atrial natriuretic peptide (pg/ml) (*n* = 13)	104.0 (50.8–163.0)	99.9 (48.7–181.0)	0.576
Antidiuretic hormone (pg/ml) (*n* = 9)	2.9 (1.4–3.5)	4.2 (1.85–4.75)	0.735

*IQR* interquartile range, *n* number, *eGFR* estimated glomerular filtration rate

U-Na and FENa increased significantly after 4 h (*p* = 0.035 and *p* = 0.022, respectively). The alteration at 8 h was more remarkable (*p* = 0.003 and *p* = 0.003, respectively). The alteration at 24 h was not significant, but FENa after 1 week significantly increased (*p* = 0.019) (Fig. 1b, c). The serum creatinine and serum sodium increased significantly after 1 week (*p* = 0.012 and *p* = 0.002, respectively), and eGFR decreased similarly (*p* = 0.008) (Table 2). However, there were 7 cases in which serum creatinine level decreased. There were no differences in blood pressure, BNP, hANP, and ADH between the baseline and 1 week with tolvaptan treatment (Table 2).

**Fig. 2:**
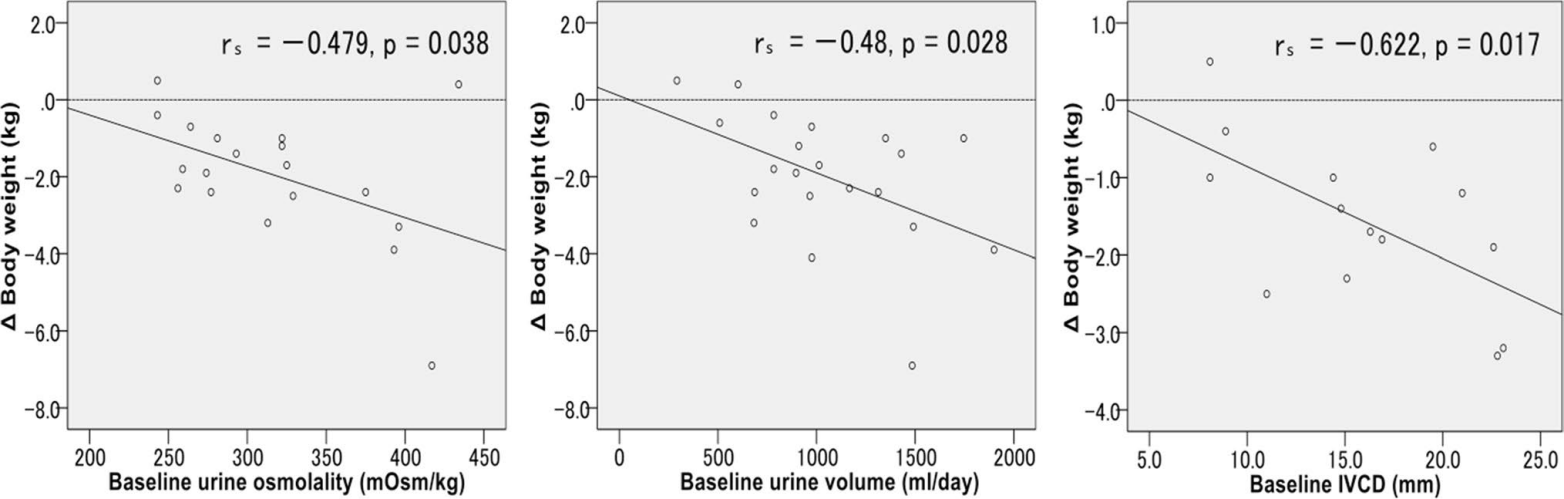
Correlations between diuretic effects of tolvaptan and body weight in CKD patients. The alteration of body weight after 1 week negatively correlated with the baseline urine osmolality (*n* = 19), urine volume (*n* = 21), and IVCD (*n* = 14)

We analyzed the relationship between body weight alteration and several parameters (Table 3). The alteration of body weight after 1 week correlated significantly with the baseline urine osmolality (*r* = −0.479, *p* = 0.038), urine volume (*r* = −0.48, *p* = 0.028), and IVCD (*r* = −0.622, *p* = 0.017) (Fig. 2). The alteration of body weight did not correlated with the baseline and after body weight. Serum creatinine, eGFR, endocrine hormone, and urea sodium excretion were not significantly correlated.

**Table 3:** Correlations of baseline parameters and body weight alteration

Parameter	*r*_s_	*p* value
Body weight (kg)	−0.098	0.672
Blood pressure (mmHg)		
Systolic	0.128	0.58
Diastolic	−0.16	0.488
Furosemide (mg/day)	−0.215	0.349
*Average urinary volume (ml/day)*	**−0.48**	**0.028**
Ejection fraction (%) (*n* = 15)	0.066	0.815
*Inferior vena cava diameter (mm) (n = 14)*	**−0.622**	**0.017**
Urine gravity (*n* = 19)	−0.376	0.113
*Urine osmolality (mOsm/kg) (n = 19)*	**−0.479**	**0.038**
U-Na (mEq/gCr) (*n* = 20)	−0.263	0.282
FENa (%) (*n* = 20)	−0.04	0.867
Serum creatinine	0.113	0.626
eGFR (ml/min/1.73 m^2^)	−0.034	0.884
Serum sodium (mEq/l)	−0.093	0.689
Serum osmolality (mOsm/kg) (*n* = 18)	−0.236	0.345
Brain natriuretic peptide (pg/ml) (*n* = 20)	−0.229	0.317
Atrial natriuretic peptide (pg/ml) (*n* = 13)	−0.412	0.162
Antidiuretic hormone (pg/ml) (*n* = 9)	−0.561	0.116

*r*_*s*_ Spearman’s rank correlation coefficient, *n* number, *U-Na* urine sodium excretion, *FENa* fractional excretion of sodium, *eGFR* estimated glomerular filtration rate

**Fig. 3:**
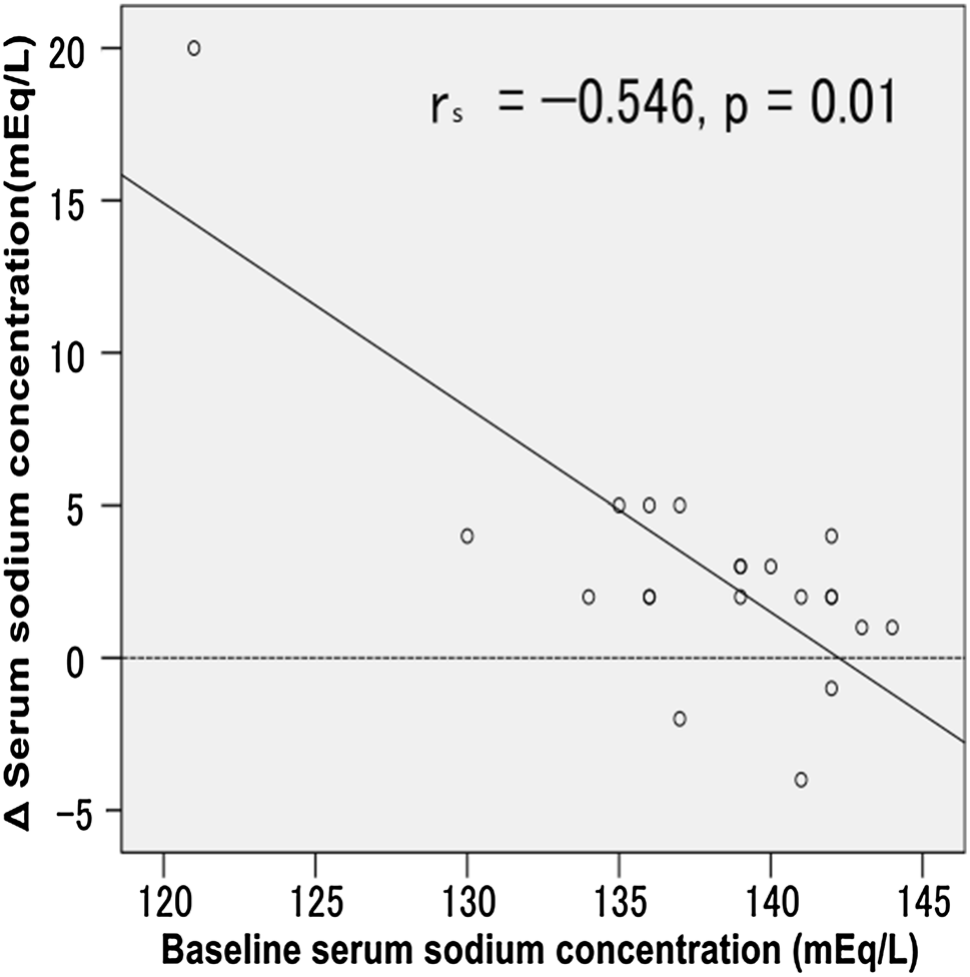
Increases in the serum sodium concentration were negatively correlated with the basal sodium levels in non-parametric test (*n* = 21)

The increases in the serum sodium concentration were negatively associated with the basal sodium levels (*r* = −0.546, *p* = 0.01) (Fig. 3). The cases of hyponatremia were improved to the normal value.

Among 21 study participants, 2 patients discontinued tolvaptan treatment during the study periods because of liver dysfunction and cerebral infarction, respectively.

## Discussion

In this study, we demonstrated that tolvaptan produced significant diuretic effects and reduced body weight in CKD patients. The urine osmolality was also decreased significantly. There was a slight increase in serum creatinine level, but improvement for hyponatremia was observed. The reduction of body weight after administration of tolvaptan was correlated with the baseline urine volume, urine osmolality, and the IVCD, but not with renal function in the study subjects.

We confirmed that body weight decreased and urine volume increased by tolvaptan in advanced stages of CKD. Although the increase of urine volumes seen in those patients were lower than those in patients with normal renal function [10, 11], it is effective to delay introducing renal replacement therapy. Even in the advanced stages of CKD, tubule-interstitial functions were reported as being maintained to a certain degree, particularly in the collecting ducts of the medullary area [12]. Collecting tubules are known as a low oxygen consumption tissue compared with the medullary thick ascending limb of Henle, which is the site of action of furosemide [13, 14], and is thought to be strong against hypoxic conditions. Thus, tolvaptan may work even if the kidney function decreased to the advanced stages of CKD. Furthermore, because tolvaptan is transported to collecting ducts without binding to proteins in blood, tolvaptan is not affected by serum or urine proteins levels as compared with furosemide [15–17]. It is also thought to be less susceptible to the glomerular filtration rate and urine flow, because it acts from the vascular side of principal cells of the collecting duct. Indeed, serum creatinine and eGFR were not correlated with diuretic effects in our study. Thus, tolvaptan may show diuretic effects in furosemide-resistant cases such as in advanced stages of CKD. Natriuretic effects of co-administering diuretics (i.e., furosemide) may also be enhanced due to the improvement of renal congestion by tolvaptan [18, 19]. Urinary excretion of sodium and FENa increased after tolvaptan treatment in our study, suggesting that furosemide resistance was improved. Moreover, plasma arginine vasopressin has stimulatory effects on epithelial Na-channel (ENaC)-mediated sodium reabsorption in the distal nephrons [20], and inhibition of V_2_ receptors by tolvaptan decreases the ENaC activity and enhances excretion of sodium in urine [21].

Recent studies reported that increased expression of aquaporin-2 was observed in the epithelial cells of the collecting ducts of tolvaptan responders [12]. However, it is still unclear what clinical factors are associated with diuretic effects and weight reduction. Therefore, we examined predictors of effects of tolvaptan, which are often measured routinely in clinical settings. We found that the baseline urine osmolality, urine volume, and IVCD negatively correlated with body weight reduction after tolvaptan treatment. Urine osmolality was reported as a predictor of distinguishing responders from non-responders to tolvaptan in decompensated heart failure patients [22]. Similarly, a significant difference in the urine osmolalities before the tolvaptan treatment was observed between responders and non-responders in CKD patients [23]. The concentrated urine suggests that the function of collecting ducts remains. Moreover, when there was no or only mild tubule-interstitial damage in the kidney biopsies, it has been reported that tolvaptan was effective even when glomerulosclerosis was present [12]. Thus, if the high urine osmolality remained, tolvaptan would demonstrate significant diuretic effects in cases with complicating renal failure. Apart from the urine osmolality, the baseline urine volume and IVCD also correlated with the alteration of body weight in this study. The high urine volume and enlarged IVCD suggest an increased intravascular fluid volume. Therefore, we estimate that diuretic effects are obtainable in these cases.

In cases of hyponatremia, the serum sodium concentration was markedly improved. Similar to our report, there was a study reporting that changes in serum sodium concentrations after tolvaptan treatment inversely correlated with their baseline values [24]. This study included a case with relative excess secretion of vasopressin in spite of hyponatremia (serum sodium 121 mEq/l, serum osmolality 258 mOsm/kg, ADH 1.0 pg/ml). Furthermore, the serum sodium markedly increased in this case; the increase of serum sodium was 20 mEq/l within 1 week. The negative correlation between baseline serum sodium concentrations and increments of serum sodium concentrations after tolvaptan treatment remained even if we excluded this case (*r* = −0.472, *p* = 0.036). Fortunately, the adverse event had not occurred in the case. However, we should pay attention to the doses of tolvaptan in the case of hyponatremia, because there is a risk of osmotic demyelination syndrome with rapid correction of serum sodium concentrations. As ADH secretion was enhanced in patients with heart failure [25], it is possible that cases with excess secretion of ADH are included in the study populations. For this reason, there may be a corrective effect for hyponatremia. Therefore, tolvaptan may be a suitable drug for combination treatments with diuretics in CKD patients with refractory chronic heart failure (CHF). On the other hand, hyponatremia was considered to be a factor of poor prognosis in cases with CKD and CHF [26], and such hyponatremic patients were known for having risks of incidental fall and bone fracture [27], which causes long-term hospitalization and deterioration of quality of life. Therefore, it is reasonable to choose tolvaptan for treating CKD with hyponatremia.

The serum creatinine concentration increased slightly in this study. However, there are several reports that tolvaptan may protect against renal function. Costello et al. reported increased renal blood flow after administration of tolvaptan among patients with heart failure as compared with placebo or furosemide [1]. It is also speculated that tolvaptan stimulates neither the sympathetic activities nor renin–angiotensin–aldosterone (RAA) systems [28]. If tolvaptan is added to the treatment, there may be the advantage of reducing the dose of furosemide, which stimulates both sympathetic and RAA systems. In some clinical studies, serum creatinine and eGFR were reported not to be altered by tolvaptan treatment [15, 18, 24, 29]. In addition, the worsening renal function risk was lower with tolvaptan than conventional treatment in patients with acute decompensated heart failure [29–31], and the effects on eGFR were similar to carperitide [32, 33]. Furthermore, it must be considered that lower eGFR was found to be a significant risk factor for a faster decline of eGFR [34]. In this study, there was a significant difference in the baseline ADH level between groups with increased and decreased serum creatinine after tolvaptan treatment (*p* = 0.048). The baseline ADH was measured in 9 cases, and the ADH level in the group with decreased serum creatinine (*n* = 3) was higher than that with increased serum creatinine level (*n* = 6). The free water retention due to excessive secreted ADH might be related to worsen renal failure in CHF, and the blockade of vasopressin V2 receptor by tolvaptan might provide improvement of the renal function.

Two cases were not able to continue use of tolvaptan because of its side effects. One patient exhibited liver dysfunction. Similarly, in the study of patients with autosomal dominant polycystic kidney disease, it was also reported that patients receiving tolvaptan had an increased risk of alanine aminotransferase or aspartate aminotransferase level elevation [35]. The other patient developed cerebral infarction. The mild hemiplegia symptom developed on the fourth day, and a small infarction lesion was detected by CT examination. There is no report of cerebral infarction associated with tolvaptan, even in the above clinical trials [35]. They were not fatal clinical conditions, but caution and accumulation of further cases are required.

This study has several limitations. As there were inconsistencies in the timing of cardiac echo (several days before administration, at the start of tolvaptan, etc), the CHF status might not be evaluated precisely. Furthermore, this is a retrospective observational study with a relatively small number of patients. It is necessary to examine the plasticity in more cases to warrant the results. A prospective study in which patients are randomized to comparison groups with placebo or other diuretics is expected to confirm our results.

In conclusion, tolvaptan has diuretic effects in cases with complicated, advanced renal dysfunction corresponding to stage 3–5 CKD, and is also effective in correcting hyponatremia. As the baseline urine osmolality, urine volume, and IVCD correlated closely with the amount of reduction in body weight, they may be useful predictors for diuretic effects of tolvaptan. In cases of maintaining urine concentrating abilities, tolvaptan may be the treatment of choice for CKD patients with fluid retention.

## References

[1] Costello-Boerrigter LC, Smith WB, Boerrigter G, Ouyang J, Zimmer CA, Orlandi C, et al. Vasopressin-2-receptor antagonism augments water excretion without changes in renal hemodynamics or sodium and potassium excretion in human heart failure. Am J Physiol Renal Physiol. 2006;290:F273–8. 10.1152/ajprenal.00195.2005 PMC2647140 16189291

[2] Yamamura Y, Nakamura S, Itoh S, Hirano T, Onogawa T, Yamashita T (1998). OPC-41061, a highly potent human vasopressin V2-receptor antagonist: pharmacological profile and aquaretic effect by single and multiple oral dosing in rats. J Pharmacol Exp Ther.

[3] Gheorghiade M, Niazi I, Ouyang J, Czerwiec F, Kambayashi J, Zampino M (2003). Vasopressin V2-receptor blockade with tolvaptan in patients with chronic heart failure: results from a double-blind, randomized trial. Circulation.

[4] Gheorghiade M, Gattis WA, O’Connor CM, Adams KF, Elkayam U, Barbagelata A (2004). Effects of tolvaptan, a vasopressin antagonist, in patients hospitalized with worsening heart failure: a randomized controlled trial. JAMA.

[5] Schrier RW, Gross P, Gheorghiade M, Berl T, Verbalis JG, Czerwiec FS (2006). Tolvaptan, a selective oral vasopressin V2-receptor antagonist, for hyponatremia. N Engl J Med.

[6] Miyazaki T, Yamamura Y, Onogawa T, Nakamura S, Kinoshita S, Nakayama S (2005). Therapeutic effects of tolvaptan, a potent, selective nonpeptide vasopressin V2 receptor antagonist, in rats with acute and chronic severe hyponatremia. Endocrinology.

[7] Felker GM, O’Connor CM, Braunwald E, Heart Failure Clinical I, Research Network (2009). Loop diuretics in acute decompensated heart failure: necessary? Evil? A necessary evil?. Circ Heart Fail.

[8] Matsuo S, Imai E, Horio M, Yasuda Y, Tomita K, Nitta K (2009). Revised equations for estimated GFR from serum creatinine in Japan. Am J Kidney Dis.

[9] Cheriex EC, Leunissen KM, Janssen JH, Mooy JM, van Hooff JP (1989). Echography of the inferior vena cava is a simple and reliable tool for estimation of ‘dry weight’ in haemodialysis patients. Nephrol Dial Transplant.

[10] Matsuzaki M, Hori M, Izumi T, Asanoi H, Tsutamoto T, Tolvaptan I (2011). Effects of tolvaptan on volume overload in Japanese patients with heart failure: results of a phase II, multicenter, randomized, double-blind, placebo-controlled, parallel-group study. Cardiovasc Drugs Ther.

[11] Matsuzaki M, Hori M, Izumi T, Fukunami M, Tolvaptan I (2011). Efficacy and safety of tolvaptan in heart failure patients with volume overload despite the standard treatment with conventional diuretics: a phase III, randomized, double-blind, placebo-controlled study (QUEST study). Cardiovasc Drugs Ther.

[12] Sato E, Nakamura T, Amaha M, Nomura M, Matsumura D, Yamagishi H (2014). Effect of tolvaptan in patients with chronic kidney disease due to diabetic nephropathy with heart failure. Int Heart J.

[13] Heyman SN, Reichman J, Brezis M (1999). Pathophysiology of radiocontrast nephropathy: a role for medullary hypoxia. Invest Radiol.

[14] Goldfarb M, Abassi Z, Rosen S, Shina A, Brezis M, Heyman SN (2001). Compensated heart failure predisposes to outer medullary tubular injury: studies in rats. Kidney Int.

[15] Tanaka A, Katsuno T, Ozaki T, Sakata F, Kato N, Suzuki Y (2015). The efficacy of tolvaptan as a diuretic for chronic kidney disease patients. Acta Cardiol.

[16] Inoue M, Okajima K, Itoh K, Ando Y, Watanabe N, Yasaka T (1987). Mechanism of furosemide resistance in analbuminemic rats and hypoalbuminemic patients. Kidney Int.

[17] Kirchner KA, Voelker JR, Brater DC (1990). Intratubular albumin blunts the response to furosemide—a mechanism for diuretic resistance in the nephrotic syndrome. J Pharmacol Exp Ther.

[18] Otsuka T, Sakai Y, Ohno D, Murasawa T, Sato N, Tsuruoka S (2013). The effects of tolvaptan on patients with severe chronic kidney disease complicated by congestive heart failure. Clin Exp Nephrol.

[19] Mullens W, Abrahams Z, Francis GS, Sokos G, Taylor DO, Starling RC (2009). Importance of venous congestion for worsening of renal function in advanced decompensated heart failure. J Am Coll Cardiol.

[20] Blanchard A, Frank M, Wuerzner G, Peyrard S, Bankir L, Jeunemaitre X (2011). Antinatriuretic effect of vasopressin in humans is amiloride sensitive, thus ENaC dependent. Clin J Am Soc Nephrol.

[21] Imamura T, Kinugawa K, Minatsuki S, Muraoka H, Kato N, Inaba T (2014). Urine sodium excretion after tolvaptan administration is dependent upon baseline serum sodium levels: a possible explanation for the improvement of hyponatremia with scarce chance of hypernatremia by a vasopressin receptor antagonist. Int Heart J.

[22] Imamura T, Kinugawa K, Minatsuki S, Muraoka H, Kato N, Inaba T (2013). Urine osmolality estimated using urine urea nitrogen, sodium and creatinine can effectively predict response to tolvaptan in decompensated heart failure patients. Circ J.

[23] Iwatani H, Kawabata H, Sakaguchi Y, Yamamoto R, Hamano T, Rakugi H (2015). Urine osmolarity predicts the body weight-reduction response to tolvaptan in chronic kidney disease patients: a retrospective, observational study. Nephron.

[24] Watanabe K, Dohi K, Sugimoto T, Yamada T, Sato Y, Ichikawa K (2012). Short-term effects of low-dose tolvaptan on hemodynamic parameters in patients with chronic heart failure. J Cardiol.

[25] Valania G, Singh M, Slawsky MT (2011). Targeting hyponatremia and hemodynamics in acute decompensated heart failure: is there a role for vasopressin antagonists?. Curr Heart Fail Rep.

[26] Kovesdy CP, Lott EH, Lu JL, Malakauskas SM, Ma JZ, Molnar MZ (2012). Hyponatremia, hypernatremia, and mortality in patients with chronic kidney disease with and without congestive heart failure. Circulation.

[27] Gankam Kengne F, Andres C, Sattar L, Melot C, Decaux G (2008). Mild hyponatremia and risk of fracture in the ambulatory elderly. QJM.

[28] Miyazaki T, Fujiki H, Yamamura Y, Nakamura S, Mori T (2007). Tolvaptan, an orally active vasopressin V(2)-receptor antagonist - pharmacology and clinical trials. Cardiovasc Drug Rev.

[29] Uemura Y, Shibata R, Takemoto K, Uchikawa T, Koyasu M, Ishikawa S, et al. Clinical benefit of tolvaptan in patients with acute decompensated heart failure and chronic kidney disease. Heart Vessels 2015. 10.1007/s00380-015-0775-9 26615607

[30] Matsue Y, Suzuki M, Seya M, Iwatsuka R, Mizukami A, Nagahori W (2013). Tolvaptan reduces the risk of worsening renal function in patients with acute decompensated heart failure in high-risk population. J Cardiol.

[31] Kimura K, Momose T, Hasegawa T, Morita T, Misawa T, Motoki H (2016). Early administration of tolvaptan preserves renal function in elderly patients with acute decompensated heart failure. J Cardiol.

[32] Suzuki S, Yoshihisa A, Yamaki T, Sugimoto K, Kunii H, Nakazato K (2013). Acute heart failure volume control multicenter randomized (AVCMA) trial: comparison of tolvaptan and carperitide. J Clin Pharmacol.

[33] Suzuki S, Yoshihisa A, Yamaki T, Sugimoto K, Kunii H, Nakazato K (2014). Long-term effects and prognosis in acute heart failure treated with tolvaptan: the AVCMA trial. Biomed Res Int.

[34] Imai E, Horio M, Yamagata K, Iseki K, Hara S, Ura N (2008). Slower decline of glomerular filtration rate in the Japanese general population: a longitudinal 10-year follow-up study. Hypertens Res.

[35] Torres VE, Chapman AB, Devuyst O, Gansevoort RT, Grantham JJ, Higashihara E (2012). Tolvaptan in patients with autosomal dominant polycystic kidney disease. N Engl J Med.

